# Structural Divergence in Vertebrate Phylogeny of a Duplicated Prototype Galectin

**DOI:** 10.1093/gbe/evu215

**Published:** 2014-09-25

**Authors:** Ramray Bhat, Mahul Chakraborty, I.S. Mian, Stuart A. Newman

**Affiliations:** ^1^Life Sciences Division, Lawrence Berkeley National Laboratory, Berkeley, California; ^2^Department of Ecology and Evolutionary Biology, University of California, Irvine; ^3^Department of Computer Science, University College London, United Kingdom; ^4^Department of Cell Biology and Anatomy, New York Medical College, Valhalla, New York

**Keywords:** prototype galectin, galectin-1, sauropsids, protein fold, homology

## Abstract

Prototype galectins, endogenously expressed animal lectins with a single carbohydrate recognition domain, are well-known regulators of tissue properties such as growth and adhesion. The earliest discovered and best studied of the prototype galectins is Galectin-1 (Gal-1). In the *Gallus gallus* (chicken) genome, Gal-1 is represented by two homologs: Gal-1A and Gal-1B, with distinct biochemical properties, tissue expression, and developmental functions. We investigated the origin of the Gal-1A/Gal-1B divergence to gain insight into when their developmental functions originated and how they could have contributed to vertebrate phenotypic evolution. Sequence alignment and phylogenetic tree construction showed that the Gal-1A/Gal-1B divergence can be traced back to the origin of the sauropsid lineage (consisting of extinct and extant reptiles and birds) although lineage-specific duplications also occurred in the amphibian and actinopterygian genomes. Gene synteny analysis showed that sauropsid *gal-1b* (the gene for Gal-1B) and its frog and actinopterygian *gal-1* homologs share a similar chromosomal location, whereas sauropsid *gal-1a* has translocated to a new position. Surprisingly, we found that chicken Gal-1A, encoded by the translocated *gal-1a*, was more similar in its tertiary folding pattern than Gal-1B, encoded by the untranslocated *gal-1b*, to experimentally determined and predicted folds of nonsauropsid Gal-1s. This inference is consistent with our finding of a lower proportion of conserved residues in sauropsid Gal-1Bs, and evidence for positive selection of sauropsid *gal-1b*, but not *gal-1a* genes. We propose that the duplication and structural divergence of Gal-1B away from Gal-1A led to specialization in both expression and function in the sauropsid lineage.

## Introduction

Galectins are proteins that bind to β-galactoside-containing glycoconjugates (Barondes et al. 1994). They are found in most metazoans where they are expressed in multiple tissues and organs, and are involved in development (Bullock et al. 2001; Bhat et al. 2011), homeostasis (Rabinovich and Croci 2012; Romaniuk et al. 2012), and disease (Gabius 2009; Lahm et al. 2004). Galectins contain one or more carbohydrate recognition domains (CRDs) as well as other domains. Prototype galectins contain a single CRD (Kaltner et al. 2008), which can either be an F3 CRD or F4 CRD depending on their exon–intron structure (Houzelstein et al. 2004). One of the best studied of the galectins is the prototype F3 CRD galectin, known as Galectin-1 (Gal-1/L-14-1; the gene encoding Gal-1 is known as *LGALS1*).

It has long been known that the *G. gallus* genome has two homologs of Gal-1: A 16-kDa lectin CG-1A (also known as CG-16, C-16 and CLL-I and which we refer to as Gal-1A) and a 14-kDa lectin CG-1B (also known as CG-14, C-14 and CLL-II, which we refer to as Gal-1B). They have distinct patterns of expression: In *G. gallus*, Gal-1A is predominantly expressed in embryonic liver and muscle, whereas Gal-1B is highly expressed in embryonic skin and intestine (Den and Malinzak 1977; Nowak et al. 1977; Beyer et al. 1980; Beyer and Barondes 1982). The two Gal-1 homologs were inferred to have diverged around the time birds split off from mammals (Sakakura et al. 1990). Given this early divergence, it is not surprising that they have distinct biochemical properties such as different isoelectric points (Beyer and Barondes 1982) and unique preferences for binding to glycan moieties (Wu et al. 2007). It is therefore reasonable to expect that they might have distinct roles in organ and organismal development. Indeed, we found that *G. gallus* Gal-1A has the ability to mediate adhesion between embryonic limb bud precartilage mesenchymal cells and is expressed very early within limb buds, where it induces skeletogenesis. In contrast, Gal-1B levels are low in limb mesenchymal cells and this galectin has no effect on their adhesion or on skeletal morphogenesis (Bullock et al. 2001; Bhat et al. 2011).

The phylogeny of divergence of the genes encoding Gal-1A and Gal-1B (*gal-1a* and *gal-1b*) was first considered about a decade and a half ago (Sakakura et al. 1990). Since then considerable new genomic information for a wide range of vertebrate species has become available. Here, we revisit the phylogenetic history of Gal-1A and Gal-1B and consider an additional issue: the extent to which Gal-1A and Gal-1B differ in their structure (and thus, potentially, function) from their nonsauropsid homologs. Tracing back their origins can provide valuable insights into the evolutionary trajectories of the lineages in which they came to be expressed.

We approached these questions from the perspectives of phylogeny, synteny and protein and DNA analysis. Our results suggest that although the gene encoding Gal-1A shifted to a new location after the origination of sauropsids, its protein was evolutionarily conserved relative to sauropsid Gal-1B. The gene encoding Gal-1B, while retaining shared synteny with nonsauropsid Gal-1s, unexpectedly diverged under positive selection. This resulted in a divergence in expression and biological roles of sauropsid Gal-1s.

## Materials and Methods

### Protein and Nucleic Acid Sequence Search

Amino acid sequences of Gal-1 and its homologs were retrieved from Ensembl (http://www.ensembl.org; Release 74), PreEnsembl (http://pre.ensembl.org; Release 66), UCSC (the University of California–Santa Cruz) Genome Browser (http://genome.ucsc.edu, last accessed October 5, 2014), GenBank (http://www.ncbi.nlm.nih.gov/genbank/, last accessed October 5, 2014), and Xenbase (http://www.xenbase.org; Version 3.0; *Xenopus tropicalis* v7.1 and *Xenopus laevis* v7.1). Sequences were retrieved by Basic Local Alignment Search Tool (BLAST)/BLAT (BLAST-like Alignment Tool) algorithm using chicken Gal-1A (ENSGALG00000011480.3) peptide sequence as input. In the case of species that overlapped between the different genome databases, amino acid sequences from non-ENSEMBL sources were compared with the Gal-1 orthologs discerned by ENSEMBL which used the Gene Orthology/Paralogy prediction pipeline (Vilella et al. 2009). In the case of one *X. laevis* homolog, whose peptide sequence was not available, the mRNA sequence from the Unigene database was conceptually translated using the Translate tool of the ExPASy portal (http://web.expasy.org/translate/, last accessed October 4, 2014). Supplementary file S1, Supplementary Material online, contains the protein sequences used in this article. We retrieved nucleotide sequences of *gal-1* genes from Ensembl (http://www.ensembl.org; Release 74) and verified by translating them using ExPASY. Sequences, their respective species, and their ID numbers are listed in supplementary file S1, Supplementary Material online.

### Terminology and Definitions

In the text and figures, we refer to a species using the binomial nomenclature, adding its common name in our first reference. Therefore, the African clawed frog is initially referred to as *X. laevis* (African clawed frog) and subsequently as *X. laevis.* Our usage of the terms orthology and paralogy was as follows: Protein orthologs are products of genes in different species that evolved from a common ancestor; protein paralogs are products of duplicated genes. Therefore, chicken Gal-1A and mouse Gal-1A are orthologs of each other; chicken Gal-1A and chicken Gal-1B are paralogs of each other. Gene names are shown italicized but not capitalized (e.g., *gal-1*). Protein names are shown with the first letter in upper case and not italicized.

### Sequence Alignment and Phylogenetic Tree Construction

Phylogenetic relationships between sequences were first inferred rapidly using the Neighborhood Joining method with MUSCLE (MUltiple Sequence Comparison by Log-Expectation) for MSA (Edgar 2004). The latter was performed with SeaView (V4.5.2) phylogenetic analysis software (Gouy et al. 2010). Subsequently, we used maximum likelihood, employing PhyML, for phylogenetic tree construction. We used LG, a model of amino acid replacement matrix with improved performance over other models such as JTT and Whelan and Goldman (Le and Gascuel 2008), and optimized for both invariant sites and across-the-tree variation in rate of evolution. Posterior branch support was computed using both approximate Likelihood Ratio test (aLRT) (Anisimova and Gascuel 2006) and bootstrap analysis (with 100 replicates). The tree searching operation was set to Nearest-Neighbor Interchange with the initial tree computed using BIONJ (Gascuel 1997, p. 776).

### Synteny Analysis

Initial searches for the location of *gal-1* genes were performed using Ensembl and UCSC genome browsers. For *X. laevis*, scaffolds were also searched on XenBase. The Genomicus Browser (Muffato et al. 2010) (http://www.dyogen.ens.fr/genomicus-70.01/cgi-bin/search.pl; version 70.01) was used to obtain a simple visual representation of gene syntenies.

### Analysis of Conservation of Residues

Multiple sequence alignments of sauropsid Gal-1As, sauropsid Gal-1Bs, mammalian, and amphibian Gal-1s were performed using MUSCLE. We then identified invariant residues (identical amino acids at the same position in all sequences), single variant residues (two functionally similar or apparently functionally dissimilar amino acids at the same position in all the sequences), double variant residues (three distinct amino acids in the same position in all the sequences), and multiple variant residues (>3 distinct amino acids per position).

The aligned string of (in)variant sites for sauropsid Gal-1As, sauropsid Gal-1Bs, mammalian Gal-1s, and amphibian Gal-1s was reconciled with the overlaid secondary structure (locations of β-strands [S1–S6b, F1–F5]).

### Fold Prediction and Comparative Analysis

The PDB files for *G. gallus* Gal-1A (1QMJ), Gal-1B (3DUI), *Homo sapiens* (human) Gal-1 (3W58), *Mus musculus* (mouse) Gal-1 (4LBQ), *Rattus norvegicus* (rat) Gal-1 (3M2M), *Bos taurus* (cow) Gal-1 (1SLT), *Rhinella arinarum* (Toad) Gal-1 (1A78), and *Conger myriaster* (Conger eel) Gal-1-1 (Congerin I) (1C1F) and Gal-1-2 (Congerin II) (1IS5) were retrieved from the RCSB Protein Data Bank (Rose et al. 2013) (http://www.rcsb.org/pdb/home/home.do, last accessed October 5, 2014). For galectins whose folds have not been determined crystallographically or spectroscopically, structure prediction was performed using PhyRE^2^ (Protein Homology/Analogy Engine) (Kelley and Sternberg 2009) (http://www.sbg.bio.ic.ac.uk/phyre2/html/page.cgi?id=index, last accessed on October 5, 2014). Briefly, the software performs an iterative sequence search of the user-supplied protein sequence using position-specific iterated BLAST (PSI-BLAST) creating statistical profiles and using them to gather progressively more distant homologs in each round. An hidden Markov model (HMM) of the family of protein sequences is constructed and aligned to several HMM models of known structures in order to generate a three-dimensional (3D) model of the input sequence. PhyRE^2^ also incorporates an ab initio folding simulation to model regions of proteins with no detectable homology to known structures using Langevin dynamics. In order to compare two protein structures to the tertiary folds of chicken Gal-1A and chicken Gal-1B, we used PDBeFold (Krissinel and Henrick 2004) (http://www.ebi.ac.uk/msd-srv/ssm/, last accessed on October 5, 2014) which employs the Secondary Structure Matching algorithm to specifically achieve the best Cα alignment of amino acids. Our metric for comparing topological similarity between the predicted models and experimentally determined structures was the *Q* score, which is computed from a formula that takes into account *N*_align_ (the maximum number of aligned residues) as well as a measure of the distance between the Cα atoms of the matched residues (RMSD) when the target and query sequences are superposed in 3D. To compare experimentally determined structures and computationally predicted folds, we computed *Q* scores for all combinations of polypeptide chains; for the crystal structures—RCSB entries 1QMJ (chicken Gal-1A) and 3DUI (chicken Gal-1B), we used coordinates with the “A” chain identifier in each PDB file. *Q* scores from alignment comparisons between two crystal structures were computed using “A” chain identifiers of both PDB files.

For two Gal-1 homologs, belonging to the early diverging mammals *Ornithorhynchus anatinus* (platypus) and *Monodelphis domestica* (opossum), it was possible to construct models without reference to the chicken Gal-1A and chicken Gal-1B tertiary folds. For the Gal-1 homologs of nonmammalian vertebrates, however, we were unable to construct homology-based tertiary fold models independent of the chicken Gal-1 structures, as the PhyRE^2^ algorithm would invariably incorporate them. We were nevertheless able to semiquantitatively assess the relative similarity of the predicted structures of Gal-1 homologs to chicken Gal-1A and Gal-1B: PhyRE^2^ ranked the folds of experimentally elucidated structures whose HMMs were closest when aligned to the HMM of the given Gal-1 homolog sequence. This ranking was based on an alignment score that takes into account residue probability distribution for each position, secondary structure similarity, and presence of insertions and deletions.

### Test for Positive Selection

The PAML4 package (Yang 2007) was used to assess whether the Gal1-B sequences may have accumulated amino acid substitutions under positive selection. Guided by the amino acid alignment, the codons of the genes were aligned in TranslatorX (Abascal et al. 2010). For the PAML4 branch site test, the branch leading to the Gal1-B lineages was designated as the “foreground branch.” The foreground branch denotes the lineage where the ratio of nonsynonymous to synonymous substitution (ω = d*N*/d*S*) is hypothesized to have increased (ω > 1) for some sites relative to the rest of the lineages. The statistical test was performed using the log-likelihood ratios obtained from the null model (no selection) and the alternative model (selection). The probability cutoff is computed based on the χ^2^ distribution with df = 1.

## Results

### The Divergence between Gal-1A and Gal-1B Can Be Traced through the Sauropsid Lineage

**Fig. 1.—:**
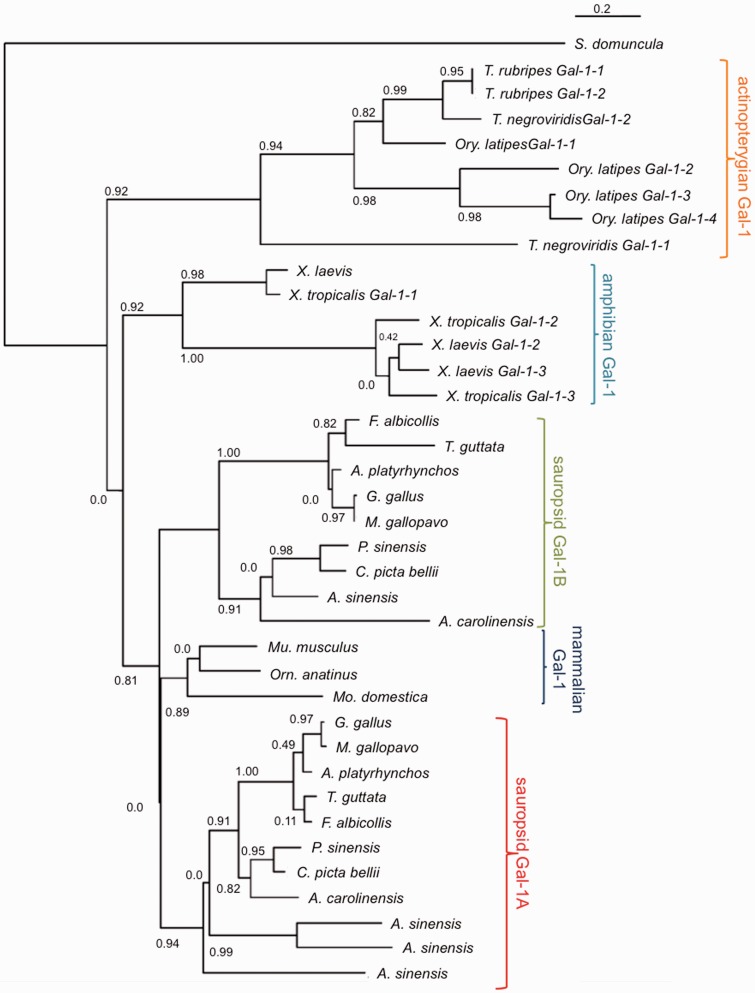
A maximum-likelihood phylogenetic tree constructed using protein sequences vertebrate Gal-1s from all orders (actinopterygians, amphibians, mammals, reptiles, and birds) with aLRT branch support shows segregation of sauropsid Gal-1As and sauropsid Gal-1Bs into separate clusters. Protein sequence for *S. domuncula* (sponge) Gal is used as an outgroup.

Homologs of Gal-1 protein from representatives of the vertebrate classes amphibia, reptilia, aves, mammalia, and actinopterygii, were used to reconstruct a maximum-likelihood phylogenetic tree using *Suberites domuncula* (sponge) as an outgroup (fig. 1) (see Materials and Methods for specific details on phylogenetic tree construction). The actinopterygian Gal-1 homologs clustered together and were separated from the tetrapod Gal-1s with strong branch support. The amphibian Gal-1 homologs also clustered with strong branch support, separate from the amniote Gal-1s, and showed an early split between *X. tropicalis* (Western clawed frog) and *X. laevis* (African clawed frog) Gal-1-1s, and Gal-1-2s and Gal-1-3s.

All examined sauropsid genomes encoded at least two homologs of Gal-1 (the genome of *Anolis carolinensis* [anole lizard] encoded four). In the phylogenetic tree, the sauropsid Gal-1 homologs showed a deep evolutionary divergence, with one homolog of every species clustering with *G. gallus* Gal-1A and the other homolog clustering with *G. gallus* Gal-1B. For convenience, we refer to a Gal-1 homolog of a given species as either Gal-1A or Gal-1B (prefixed by the species name) on the basis of whether it occurs with chicken Gal-1A or Gal-1B in the phylogenetic tree. The mammalian Gal-1s (belonging to all three orders) cluster on their own on a branch after the sauropsid Gal-1Bs split off from them and sauropsid Gal-1As, although there is no support for the branch that separates mammalian Gal-1s and sauropsid Gal-1As from sauropsid Gal-1Bs. To probe this further, we reconstructed two trees, one using NJ method and the other using maximum-likelihood using only amniotes as the sampled taxon (supplementary figs. S1 and S2, Supplementary Material online). We found that the clustering of mammalian Gal-1s, sauropsid Gal-1As, and sauropsid Gal-1Bs in both trees was topologically similar to figure 1, the main difference being that the support for the branch containing mammalian Gal-1 and sauropsid Gal-1A was stronger. The phylogenetic reconstructions therefore suggest that the genes encoding Gal-1A and Gal-1B diverged around the origin of sauropsids. It also indicates that the duplications that took place in the amphibian and actinopterygian classes are lineage-specific and independent of the sauropsid Gal-1A/Gal-1B divergence.

### *gal-1b* and Nonsauropsid gal-1 Share Synteny: *gal-1a* Occupies a New Locus Conserved across Sauropsids

**Fig. 2.—:**
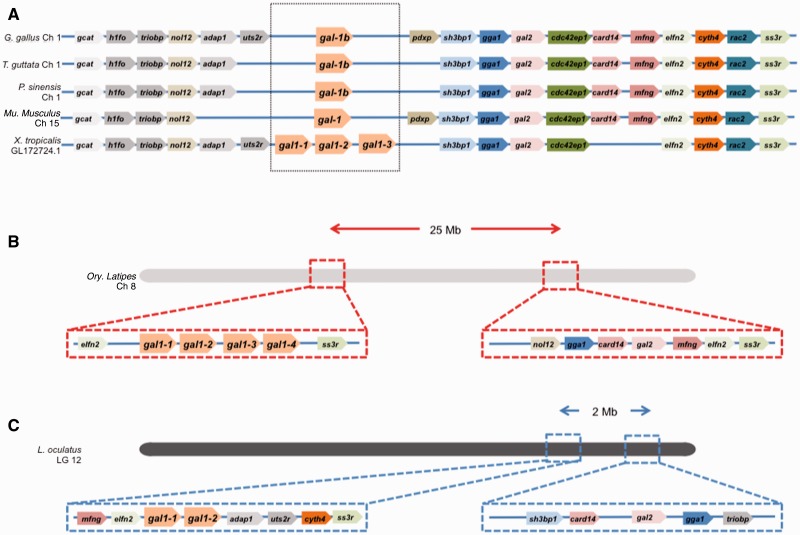
Synteny of the *gal-1b* gene and its vertebrate homologs. (*A*) Syntenic relationship between *G. gallus gal-1b* and its flanking genes with their respective homologs in *T. guttata*, *P. sinensis*, *Mu. musculus*, and *X. tropicalis* genomes. (*B*) Schematic depiction of *Ory. latipes* chromosome 8 showing that the two distinct gene loci containing *gal-1a* and *gal-2* are separated by 25 Mb. (*C*) Schematic depiction of spotted gar chromosome LG12 showing that the two distinct gene loci consisting of *gal-1a* and *gal-2* are separated by 2 Mb.

An examination of the synteny of the *gal-1* homologs across vertebrate genomes reveals genomic locations with different extents of conservation. In every sauropsid genome examined, the genomic location of *gal-1b* was conserved with respect to the neighboring genes (for convenience, we call this the *gal-1b* position) (fig. 2*A*). In the genome of the amphibian *X. tropicalis*, all three *gal-1* homologs reside at this location, as does at least one Gal-1 homolog of every mammalian genome examined. Interestingly, one of the genes found to reside close to *gal-1b* in all tetrapods is *gal-2* (with a distance of 40 kb in *G. gallus*, 90 kb in *Pelodiscus sinensis* [Chinese softshell turtle], and 150 kb in *X. tropicalis*; see also Mehrabian et al. 1993). This does not hold for the nontetrapod actinopterygii however, where for example, the *Oryzias latipes* (medaka) genome has all four of its *gal-1* homologs flanked by *elfn2* (extracellular leucine rich fibronectin domain 2) on one side and *ss3r* (somatostatin receptor 3) on the other, and approximately 25 Mb downstream, its *gal-2* is flanked by most of the other genes belonging to the tetrapod *gal-1b* location (fig. 2*B*). *ss3r* is also present adjacent to the *Ory. latipes gal-2* locus, suggesting that the two loci were generated as a result of the duplication that took place in the teleost lineage (Opazo et al. 2013). Prior to the duplication event, *gal-1* and *gal-2* were much closer (2 Mb apart in the earlier diverging *Lepisosteus oculatus* [spotted gar]) although nowhere as close as in tetrapods. The spatial relationship between *gal-1* and *gal-2* is indicative of the fact that the synteny of genes near *gal-1b* in the amphibian genome is conserved throughout the tetrapod clade, and different from that in any actinopterygian genome (fig. 2*C*). This suggests further that the tetrapod *gal-1b* genomic location has been conserved since the time tetrapods split off from the actinoptergygii.

**Fig. 3.—:**
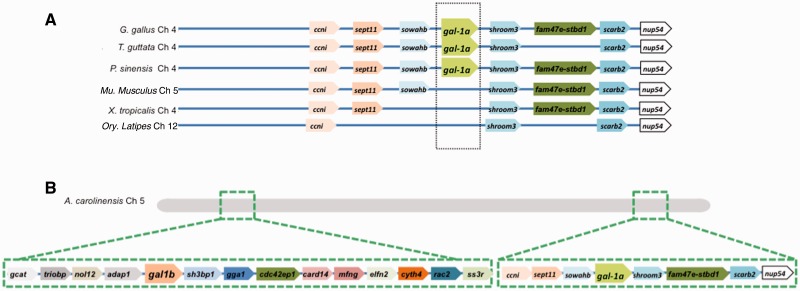
Synteny of the *gal-1a* gene and its vertebrate homologs. (*A*) Syntenic relationship between chicken *gal-1a* and its flanking genes with their respective homologs in *T. guttata*, *P. sinensis*, *Mu. musculus*, *X. tropicalis*, and *Ory. latipes* genomes. (*B*) Schematic depiction of anole lizard chromosome 5 showing the two distinct gene loci containing *gal-1a* and *gal-1b* on the same chromosome.

The genomic location of *G. gallus gal-1a* and its neighbors, *shroom3* and *sowahb* (sosondowah ankyrin repeat domain family member B), in Chromosome 4 is also conserved across the sauropsid clade (fig. 3*A*), and is hence referred to as the *gal-1a* location. In the *X. tropicalis* genome, although all the neighbors of sauropsid *gal-1a* are present in proximity to one another, this genomic location contains no *gal-1* homolog. The same holds for the actinopterygian and mammalian genomes. It is worth noting that with one exception, in all the sauropsid genomes examined, the *gal-1a* locus and the *gal-1b* locus are on different chromosomes; in the *A. carolinensis* genome, in contrast, these two loci are on the same chromosome (Chr 5) (fig. 3*B*). The fact that *gal-1a* and *gal-1b* in the genomes of turtles, birds, and *Alligator sinensis* (alligator) show greater similarity to one another in sequence and synteny than to their *A. carolinensis* homologs is consistent with the earlier suggestion that the testudines (turtles) are closer to the archosauromorphs (crocodiles, birds, and their extinct relatives) than to the lepidosauromorphs (lizards, snakes, and their extinct relatives) (Hedges and Poling 1999). Our syntenic analysis therefore shows that the *gal-1b* position is the conserved genomic location of *gal-1* in vertebrates and evolutionarily much older than the *gal-1a* position, which is present only in sauropsids. These findings led us to consider the nature and functional consequence of the duplication that gave rise to Gal-1A and Gal-1B. Did the divergence lead to an corresponding sequence-/structure-level divergence of these isoforms from their presauropsid Gal-1 homologs?

### Comparison of Gal-1 Protein Folds Shows Affinity between Nonsauropsid Gal-1 and Sauropsid Gal-1A

The biological role of a protein is a function of the 3D structure achieved by the folding of its polypeptide chain. Identification of possible conservation at the level of relevant secondary structure signatures by a residue-by-residue comparison between *G. gallus* Gal-1 isolectins and their homologs in other vertebrate classes should ideally be performed on X-ray crystallographic structures of the vertebrate galectins. Where such data are not available, as for many of these proteins, comparisons using other information can be instructive. For example, the Cys3–Cys8 residue combination was noted to be a structural feature that distinguishes *G. gallus* Gal-1B from *G. gallus* Gal-1A (Lopez-Lucendo et al. 2009). Cys 8 does not lie within the core ligand-binding site (F4-S6a/S6b) of *G. gallus* Gal-1B but within its dimer interface. An interdomain disulfide bond exists in CG-1B and may underlie its capability (in contrast to CG-1A) of becoming an oxidized covalently linked dimer (Lopez-Lucendo et al. 2004). Moreover, an intradomain disulfide bond can also form between Cys 3 and Cys 8. We found that Cys 8 is present in all sauropsid Gal-1Bs (Cys 3 is present in all sauropsid Gal-1Bs except that of *Chrysemys picta belli* [painted turtle], where it is replaced by Ser) (supplementary fig. S3, Supplementary Material online). In contrast, Cys 8 is neither found in any sauropsid Gal-1A, nor in any amphibian, mammalian or actinopterygian Gal-1 homolog.

**Fig. 4.—:**
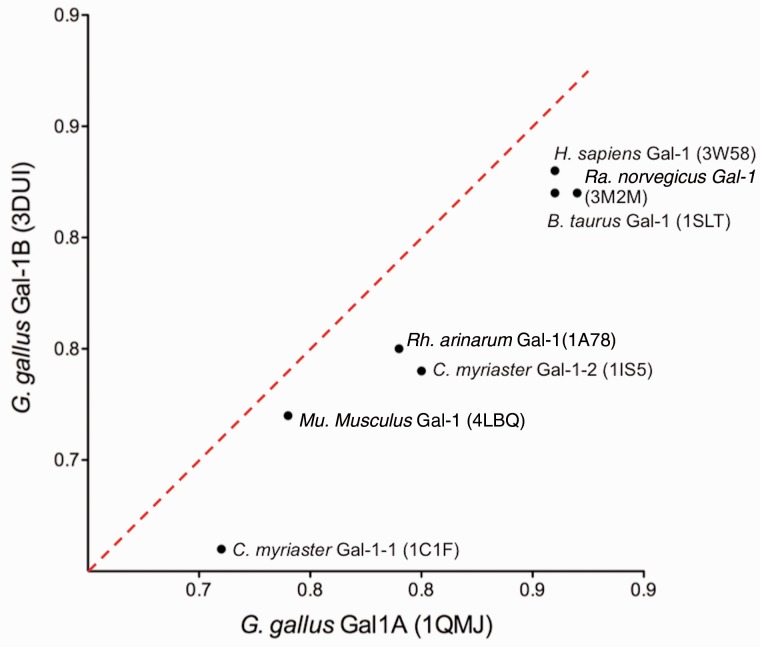
Dot plot showing of alignment (*Q* scores) of each experimentally determined fold compared with the experimentally determined fold of *G. gallus* Gal-1A (*x* axis) and *G. gallus* Gal-1B (*y* axis).

We next compared the tertiary structures of Gal-1 homologs that have been experimentally determined: *H. sapiens* (human), *Mu. musculus* (mouse), *Ra. norvegicus* (rat), *B. taurus* (cow), *Rh. arenarum* (toad), and *C. myriaster* (Conger eel) (supplementary table S1, Supplementary Material online), with those of *G. gallus* Gal-1A and Gal-1B (fig. 4). A dot plot with *Q* scores of each Gal-1 homolog with *G. gallus* Gal-1A and with *G. gallus* Gal-1B showed higher correlation of all compared homologs with the chicken Gal-1A fold than with the chicken Gal-1B fold. The computationally predicted models of the folds of both *Mo. domestica* and *Orn. anatinus* Gal-1s showed higher correlation with chicken Gal-1A fold relative to Gal-1B fold (supplementary table S2, Supplementary Material online). Moreover when all sauropsid Gal-1As, amphibian Gal-1s, and actinopterygian Gal-1s were compared with a library of experimentally determined folds, the rank for their proximity to the chicken Gal-1A fold was higher than the rank for their proximity to the chicken Gal-1B fold. On the other hand, nonchicken avian and turtle Gal-1Bs ranked as more similar to the chicken Gal-1B fold (supplementary table S3, Supplementary Material online). Interestingly, Gal-1B folds of alligator and anole lizard ranked closer to the chicken Gal-1A fold.

### Sauropsid *gal-1b* May Have Undergone Positive Selection; Sauropsid *gal-1a* Did Not

The findings on folding patterns and Gal-1 phylogeny (fig. 1) led us to hypothesize that *gal-1b,* following the duplication event, might have accumulated amino acid substitutions that led to its functional divergence with respect to the ancestral *gal-1*. Strong support for this hypothesis came from our comparison of shared invariant, single variant, double variant, and multivariant amino acid sites within sauropsid Gal-1As, sauropsid Gal-1Bs, mammalian Gal-1s, and amphibian Gal-1s. The proportion of sites with invariant and single variant residues in sauropsid Gal-1As, mammalian Gal-1s, and amphibian Gal-1s was much higher than the proportion of sites showing two or more distinct residues. In contrast, sauropsid Gal-1B showed a relatively lower proportion of sites with invariant and single variant residues and a higher proportion of sites with more than two residues (table 1). Moreover, several variable sites for sauropsid Gal-1B were concentrated together in specific domains such as in the turn between S3 and S4 strands, within and between the F3 and F4 strands, in the S2 strand, and in the C-terminus (supplementary fig. S4, Supplementary Material online). The other three sets showed relatively lower variance at these locations, instead showing notable variance in and around the S6a and S6b strands (and in case of amphibian Gal-1s, in the N-terminus).

**Table 1:** Number of Sites with Shared Invariant, Single Variant, Double Variant and Multiple Variant Residues, as well as the Collective Proportion of Invariant and Single Variant, and of Double and Multiple Variant Residues in Each of the Subsets of Sauropsid Gal-1As, Sauropsid Gal-1Bs, Mammalian Gal-1s, and Amphibian Gal-1s Are Listed

Taxon-Specific Gal Sets	Invariants	Single Variants	Percentage of Invariants + Single Variants (%)	Double Variants	Multiple Variants	Percentage of Double Variants + Multiple Variants (%)
Sauropsid Gal-1A	49	57	81	18	7	19
Sauropsid Gal-1B	40	46	64	25	24	36
Mammalian Gal-1	69	41	82	17	8	18
Amphibian Gal-1	46	54	76	25	8	24

Could *gal-1b* have, therefore, diverged under positive selection? Evidence for this is often taken as an indication of functional divergence (Zhang et al. 1998). The branch site test in the PAML4 package (Yang 2007) facilitates investigation of this hypothesis by testing whether the rate of nonsynonymous substitution along a specified lineage, termed the foreground branch, had increased under positive selection. Given the *gal-1* phylogenetic tree (fig. 1) and using the basal branch leading to *gal-1b* as the foreground branch, the PAML4 branch site test revealed that 6% of the Gal-1B amino acid sites, which are either neutral (ω = 1) or evolving under purifying selection (ω < 1) in the background branches, have accumulated nonsynonymous substitutions under historical positive selection (supplementary table S4, Supplementary Material online) (log likelihood ratio test, χ^2 ^= 4.4, df = 1, *P* < 0.05). The same test when performed for *gal-1a* did not show any statistical evidence for positive selection (log-likelihood ratio test, χ^2 ^= 1.06, df = 1, *P* > 0.05). Therefore, our analysis based on rates of synonymous and nonsynonymous substitution rates supports the hypothesis that sauropsid *gal-1b* may have diverged functionally with respect to the presauropsid *gal-1* under positive selection. In contrast, sauropsid *gal-1a* did not show any sign of having evolved under positive selection.

Apart from detecting the signatures of positive selection in amino acid sequence, the branch site test also facilitates identification of the amino acid sites that might have been targets of positive selection in the foreground branches. For Gal-1B, the branch site test identified the residue Cys8 as the target of selection (ω > 1) at the 3% and 6% level of significance in Naïve and Bayes Empirical Bayes analysis, respectively (Zhang et al. 2005). This suggests, independently of the evidence based on the crystal structures of Gal-1A and Gal-1B, that Cys8 might have played an important role in functional divergence between Gal-1A and Gal-1B.

## Discussion

By comparing protein sequences and syntenic regions, we have shown that the divergence between Gal-1A and Gal-1B, the two functionally specialized homologs of the prototype galectin Gal-1, extends across the sauropsid clade. Actinopterygian and amphibian genomes show lineage-specific duplications of Gal-1. Mammalian genomes mostly have a single Gal-1, which clusters (albeit weakly) with sauropsid Gal-1As in the phylogenetic tree and shares structure-level affinities with sauropsid Gal-1As. It is striking that Gal-1s of mammals, amphibians, and actinopterygii mostly bear sequence and structure-affinities with sauropsid Gal-1As, whereas their genes share synteny with sauropsid *gal-1b* and not *gal-1a*. Based on comparison of shared invariant amino acid sites, and especially the rates of synonymous and nonsynonymous site substitution, we also infer that sauropsid *gal-1b* may have evolved under positive selection in contrast to sauropsid *gal-1a*, which remained relatively conserved with respect to their presauropsid homologs. Our findings suggest that *gal-1* remained at a particular genomic location at least until the mammals split off from the bird–reptile lineage, after which it underwent duplication into *gal-1a* and *gal-1b* in the sauropsids.

Strikingly, subsequent to the duplication, *gal-1a* moved to a new genomic location but continued to specify the ancestral-type fold, whereas *gal-1b* remained at the presauropsid location but underwent structural divergence from both presauropsid *gal-1* and sauropsid *gal-1a*. Although this situation appears to be unusual, functional diversification of protein with the retention of the ancestral function by a relocated duplicated gene has previously been reported in a bacterial system (Ponomarev et al. 2003). Eutherian *gal-1*s have undergone negative selection, especially in their glycan binding and dimerization sites, presumably in order to conserve physiological functions such as immune tolerance at the maternal–fetal interface across placental animals (Than et al. 2008). We show here that sauropsid *gal-1b* underwent positive selection leading to the emergence of a tertiary fold in its translated protein relatively dissimilar from sauropsid Gal-1A and nonsauropsid Gal-1 fold. The acquisition of this new fold could have potentially led to novel physiological or developmental functions (neofunctionalization).

We note, moreover, that gene duplication is also one of the best-established mechanisms for subfunctionalization (partitioning of functions of the prior ancestral gene) of paralogous genes (Ohno 1970; Hahn 2009). The limited information on the functions of *gal-1a* and *gal-1b*, as well as for most presauropsid *gal-1s*, makes it difficult to assess whether the duplications of gal-1 resulted in subfunctionalization or neofunctionalization. Our analysis of tertiary structure evolution of vertebrate galectins suggests acquisition of a new folding pattern function in sauropsid Gal-1Bs. On the other hand, an analysis of tissue- and organ-specific gene expression in chickens by Sakakura et al. (1990) shows an overlap in expression patterns of *gal-1a* and *gal-1b* in *G. gallus*: stomach and muscle both express *gal-1a* and *gal-1b* and brain, liver, and heart only express *gal-1a*. In addition, expressed sequence tag determination indicates expression of *gal-1* in muscle and stomach of mammals (*Mu. musculus* [UGID:270659], *H. sapiens* [UGID:24038]) and amphibians (*X. tropicalis* [UGID:1265194]) and of a *gal-1*-like gene in *Salmo salar* (salmon) (UGID:3025170). Taken together, these observations suggest that subfunctionalization in expression has taken place after the divergence of Gal-1A and Gal-1B. In order to reconcile these two observations, we propose that the Gal-1A and Gal-1B homologs in the sauropsid lineage underwent “specialization,” a process that involves both neofunctionalization and subfunctionalization of duplicated paralogs (Conrad and Antonarakis 2007; Innan 2009; Assis and Bachtrog 2013). Although the evolutionary divergence between presauropsid Gal-1s and sauropsid Gal-1Bs is associated with differences in tertiary structure, the divergence between sauropsid Gal-1As and sauropsid Gal-1Bs and their nonsauropsid homologs may be reflected in differences in organ-specific gene expression. Gene duplication and divergence by specialization is typically accompanied by positive selection on the protein sequence of at least one of the paralogs (Hahn 2009), as appears to have occurred with the sauropsid *gal-1b* genes.

Interestingly, the Bgee Gene Expression Evolution database (http://bgee.unil.ch, last accessed October 5, 2014) shows elevated expression levels of *gal-1* in the embryonic fin buds of the zebrafish *Danio rerio*, the limb buds of metamorphosing *X. laevis* tadpoles, and in embryonic fore- and hind-limb buds of *Mu. musculus* (Bastian et al. 2008). In chicken embryonic limb buds, we found markedly elevated levels of only Gal-1A but not Gal-1B (Bhat et al. 2011). In the latter study, Gal-1A functioned in skeletogenesis by its incorporation into a regulatory network with another galectin, Gal-8, which in concert with it determined its quasiperiodic pattern of expression (Bhat et al. 2011; Glimm et al. 2014). This may be a case of a structurally conserved protein with an ancestral function (preskeletal mesenchymal condensation, seen even in fish; Eames et al. 2012), acquiring novel developmental expression patterns by coming under a new regulatory regime. Indeed, we have preliminary evidence for a conserved noncoding motif of several dozen base pairs syntenically associated with many tetrapod *gal-1* homologs and that in coelacanth, but in no actinopterygian fish (RB and SAN, in preparation). Although whole organ expression levels do not permit a definitive evaluation of developmental expression between species, future efforts will analyze the spatial expression of Gal-1 in fin- and limb-mesenchyme of actinopterygian, sarcopterygians, and amphibians with the aim of delineating possible changes in the functional roles of Gal-1 in the fin–limb transition.

## Supplementary Material

Supplementary file S1, figures S1–S4, and tables S1–S4 are available at *Genome Biology and Evolution* online (http://www.gbe.oxfordjournals.org/).

Supplementary Data
