# Supplementary material for: Structural Divergence in Vertebrate Phylogeny of a Duplicated Prototype Galectin
Source: Genome Biol Evol. 2014 Sep 25;6(10):2721–30. doi: 10.1093/gbe/evu215 (PMC4224342; doi:10.1093/gbe/evu215)
Supplement: Supplementary Data [file supp_6_10_2721__index.html]

Structural Divergence in Vertebrate Phylogeny of a Duplicated Prototype Galectin — Supplementary Data 

# Structural Divergence in Vertebrate Phylogeny of a Duplicated Prototype Galectin

## Supplementary Data

files

**Files in this Data Supplement:**

- Supplementary Data - docx file
- Supplementary Data - tif file
- Supplementary Data - tif file
- Supplementary Data - tif file
- Supplementary Data - tif file
- Supplementary Data - tif file
- Supplementary Data - tif file
- Supplementary Data - tif file
- Supplementary Data - tif file
