# Supplementary material for: Structural Divergence in Vertebrate Phylogeny of a Duplicated Prototype Galectin
Source: Genome Biol Evol. 2014 Sep 25;6(10):2721–30. doi: 10.1093/gbe/evu215 (PMC4224342; doi:10.1093/gbe/evu215)
Supplement: Supplementary Data [file supp_evu215_Supplementary_figure_captions.docx]

**Supplementary File 1** (.txt file)

List of vertebrate Gal-1 proteins used in this study along with their amino acid sequences and their Ensembl/Uniprot IDs

**Supplementary Figure Legends**

Table S1: Table showing Q-scores of alignment of experimentally determined folds of Gal-1 from mammals, toad and Conger eel, with the folds of *G. gallus* Gal-1A and *G. gallus* Gal-1B. The PDB identifier codes are included within parentheses for each fold. Higher values are highlighted in red.

Table S2: The upper table shows Q-scores of alignment of computationally predicted Gal-1 folds of *M. domestica* and *O. anatinus* with the experimentally determined folds of *G. gallus* Gal-1A and *G. gallus* Gal-1B. The lower table shows the PDB codes of the known protein folds that PHYRE^2^ deployed in order to construct the predicted tertiary folds of *M. domestica* and *O. anatinus* Gal-1.

Table S3: Rank number of chicken Gal-1A and chicken Gal-1B folds when compared using PHYRE^2^ on the basis of aligned residues and quality of alignment with sauropsid Gal-1As, sauropsid Gal-1Bs, amphibian and actinopterygians Gal-1s. Higher rank is highlighted in red.

Table S4: dN/dS for different site classes (Zhang et al. 2005) in Gal-1. The Gal-1B lineage represents the foreground branch (* = p<0.05).

Figure S1: A neighborhood joining phylogenetic tree constructed using protein sequences vertebrate Gal-1s from amniotes with bootstrap support (n=100,000) shows segregation of sauropsid Gal-1As and sauropsid Gal-1Bs into separate clusters. The sponge *S. domuncula*, which has a single galectin, is used as an outgroup.

Figure S2: A maximum-likelihood phylogenetic tree constructed using protein sequences of Gal-1s from amniotes with bootstrap support (n=100) shows segregation of sauropsid Gal-1As and sauropsid Gal-1Bs into separate clusters. *S. domuncula* is used as an outgroup.

Figure S3: Multiple sequence alignment of the N-terminal region of vertebrate Gal-1A homologs overlaid with secondary structure (shown above the sequences) showing conservation of cysteine pairs (highlighted in blue) among vertebrate Gal-1 homologs.

Figure S4: Alignment of amino acid sites with invariants and single variants shown in yellow and double and multiple variants shown in gray that are shared within sauropsid Gal-1A, sauropsid Gal-1B, mammalian Gal-1 and amphibian Gal-1, when each set is aligned using MUSCLE.
