# Supplementary figures and images for: Structural Divergence in Vertebrate Phylogeny of a Duplicated Prototype Galectin
Source: Genome Biol Evol. 2014 Sep 25;6(10):2721–30. doi: 10.1093/gbe/evu215 (PMC4224342; doi:10.1093/gbe/evu215)

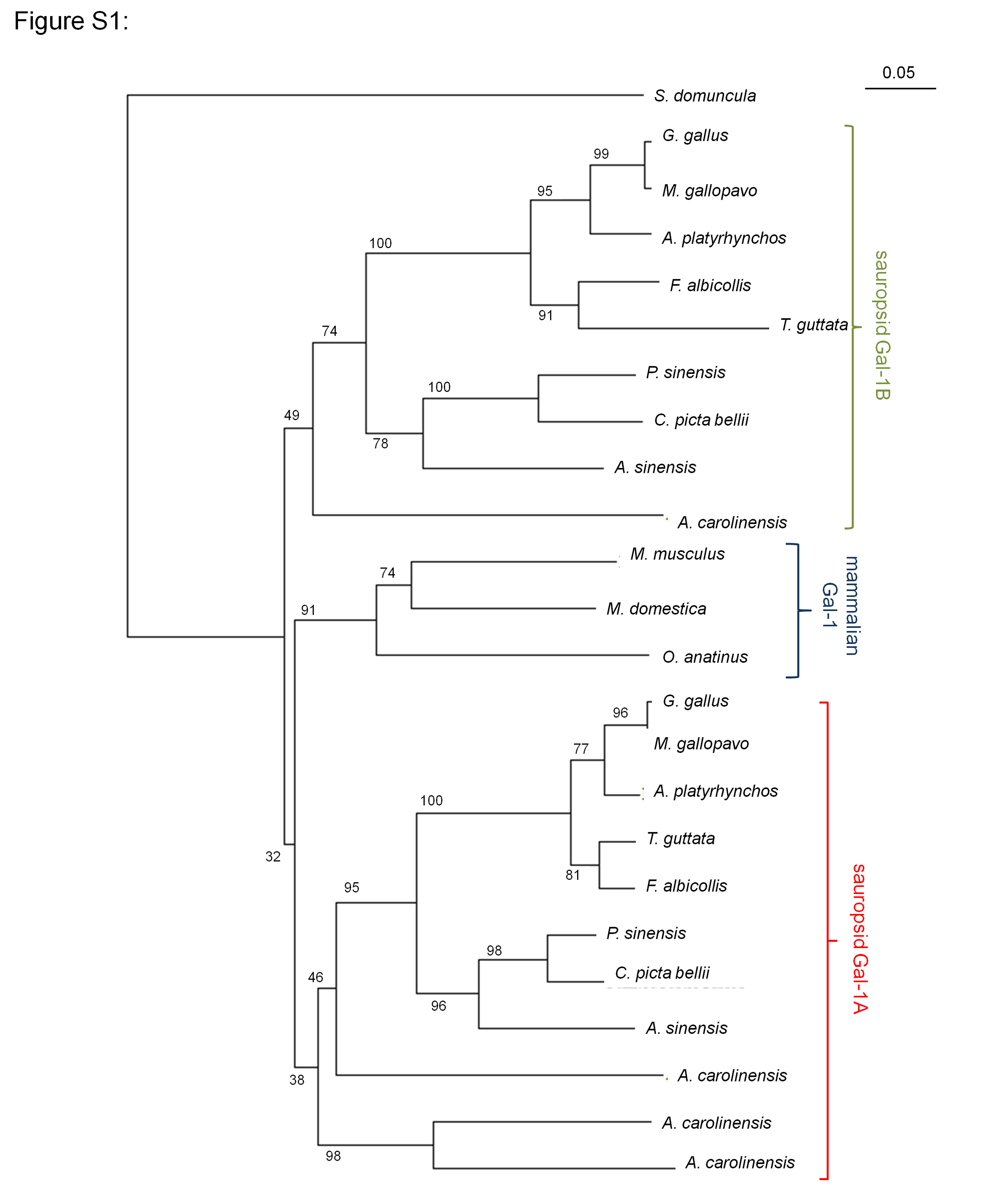

Supplement: Supplementary Data [file supp_evu215_Figure_S1.tif]

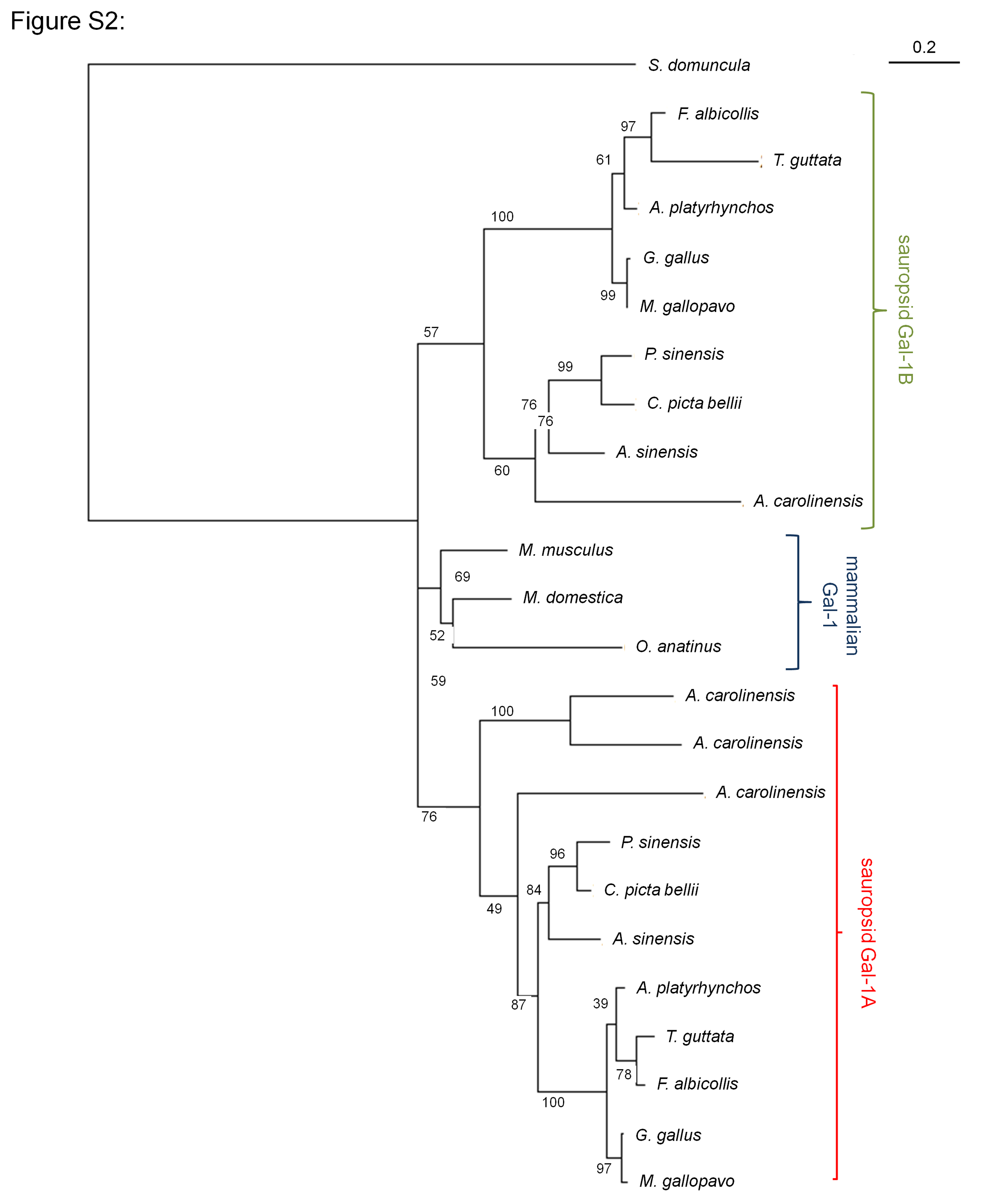

Supplement: Supplementary Data [file supp_evu215_Figure_S2.tif]

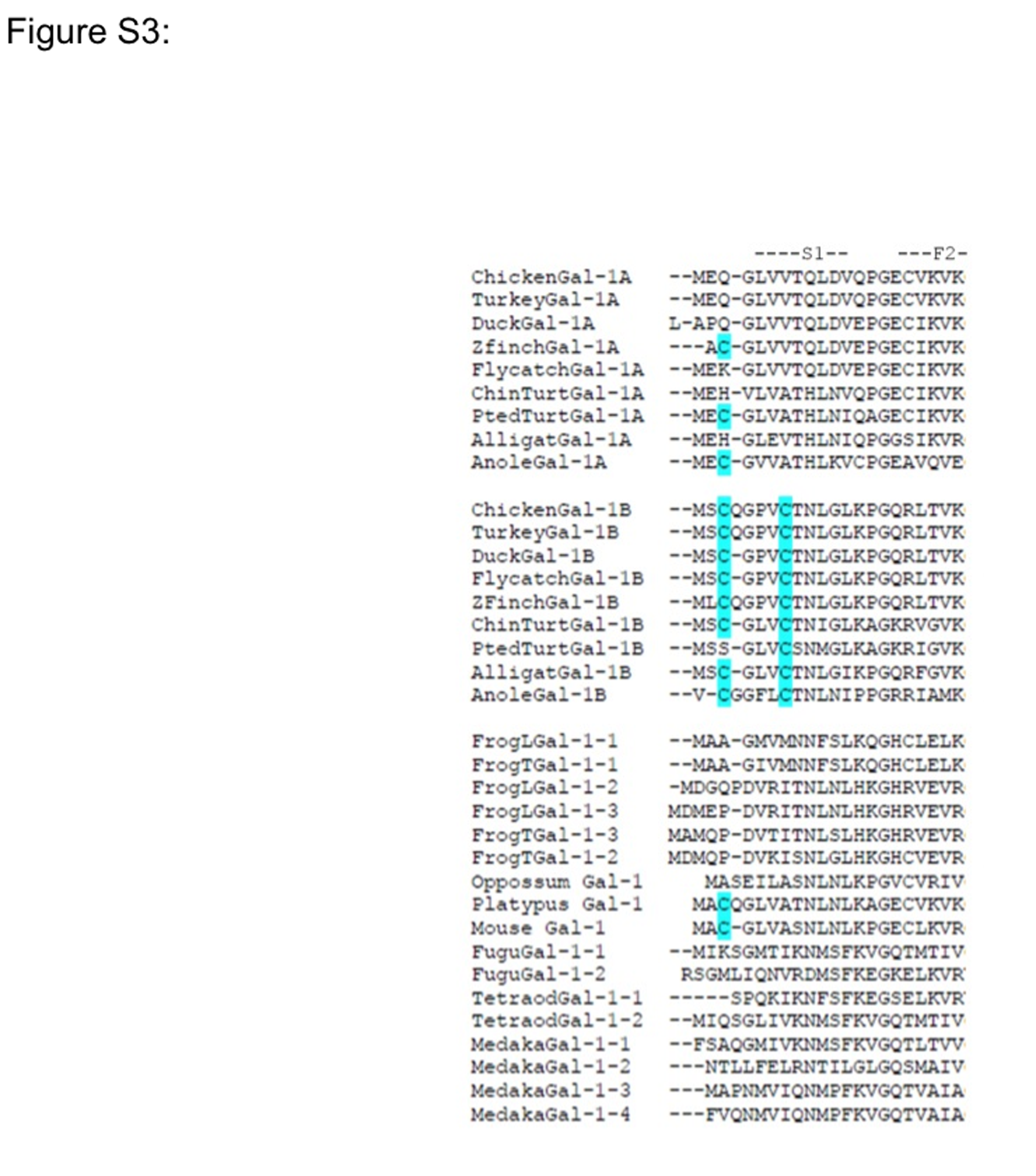

Supplement: Supplementary Data [file supp_evu215_Figure_S3.tif]

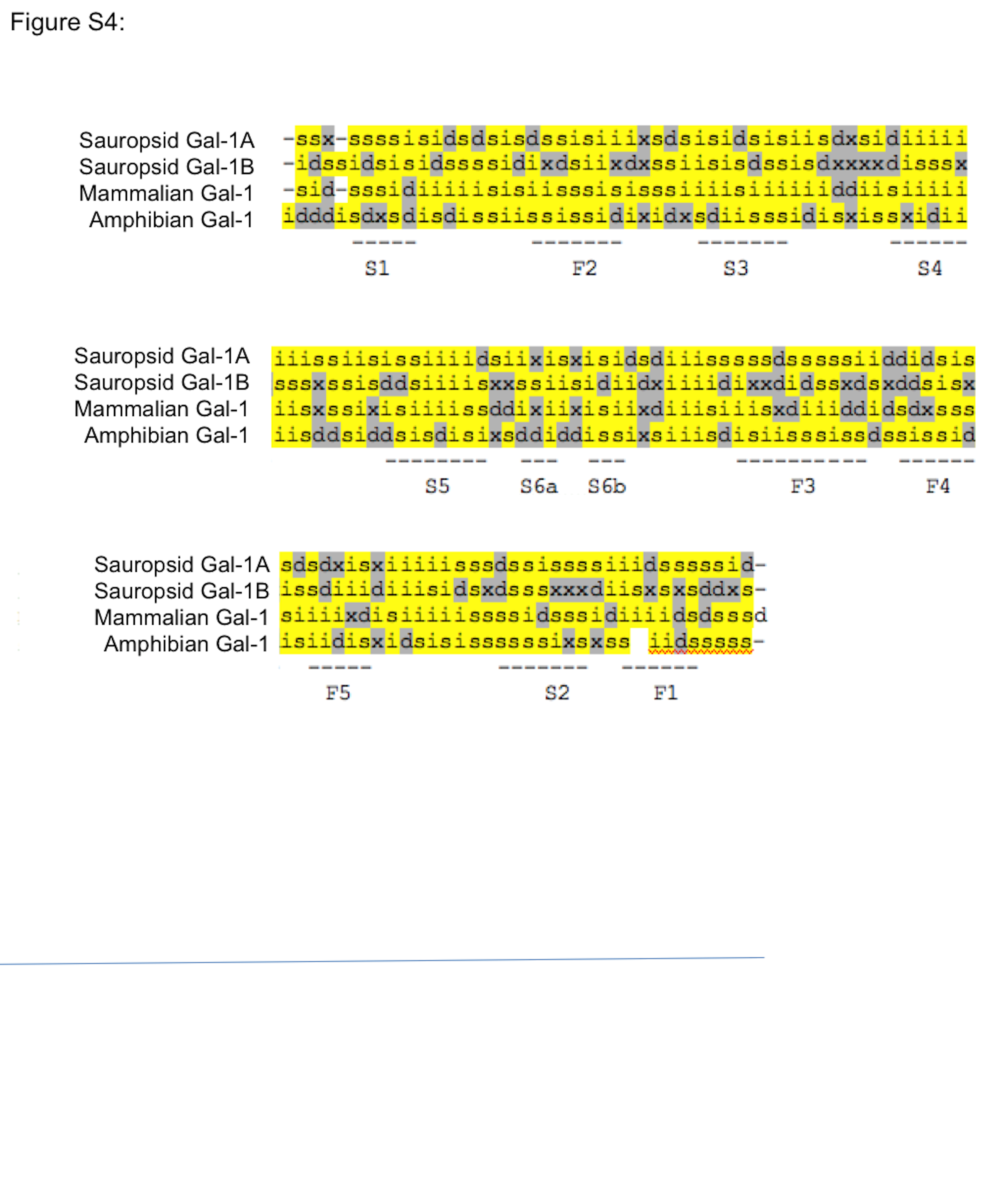

Supplement: Supplementary Data [file supp_evu215_evu215-Figure_S4.tif]

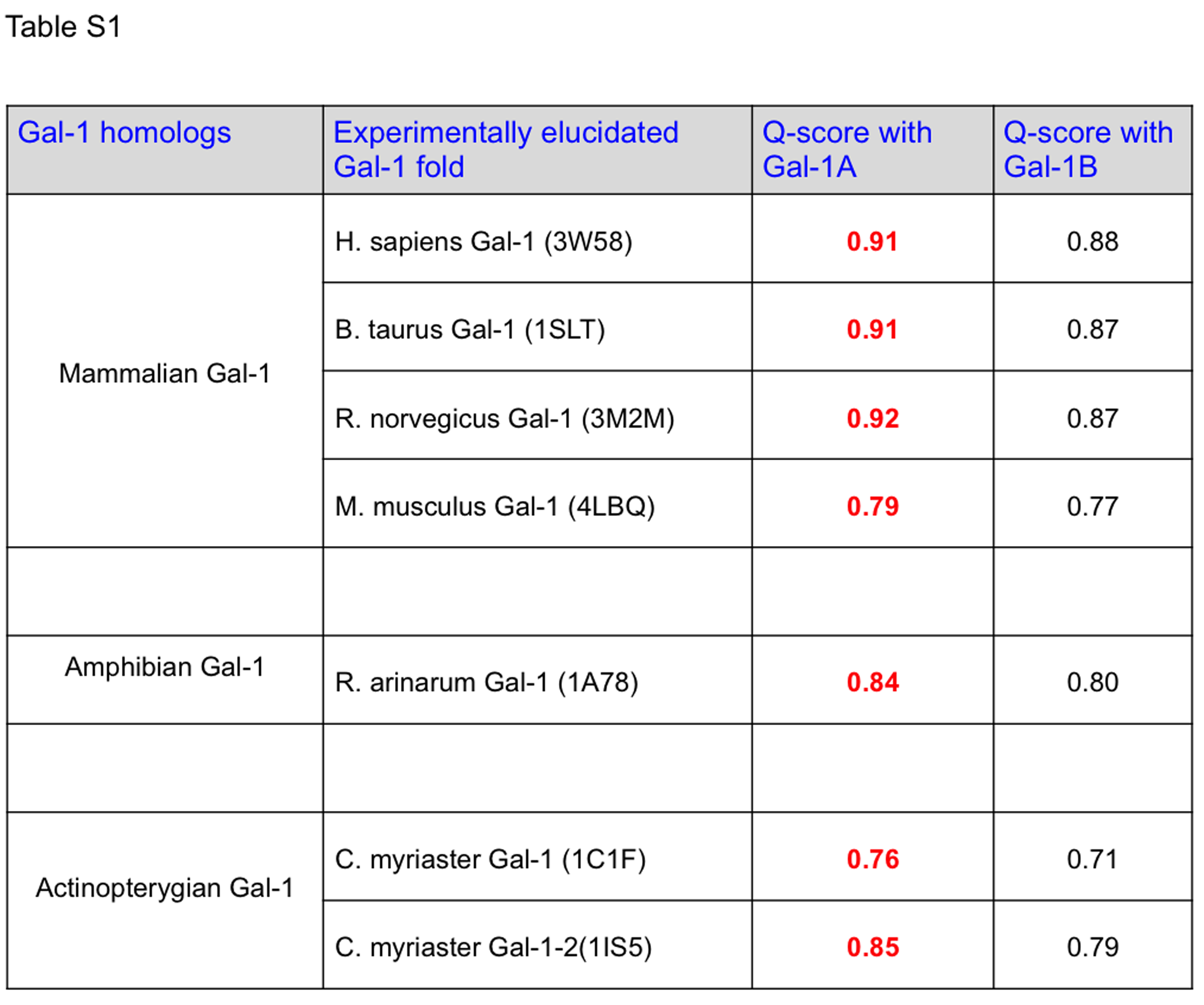

Supplement: Supplementary Data [file supp_evu215_Table_S1.tif]

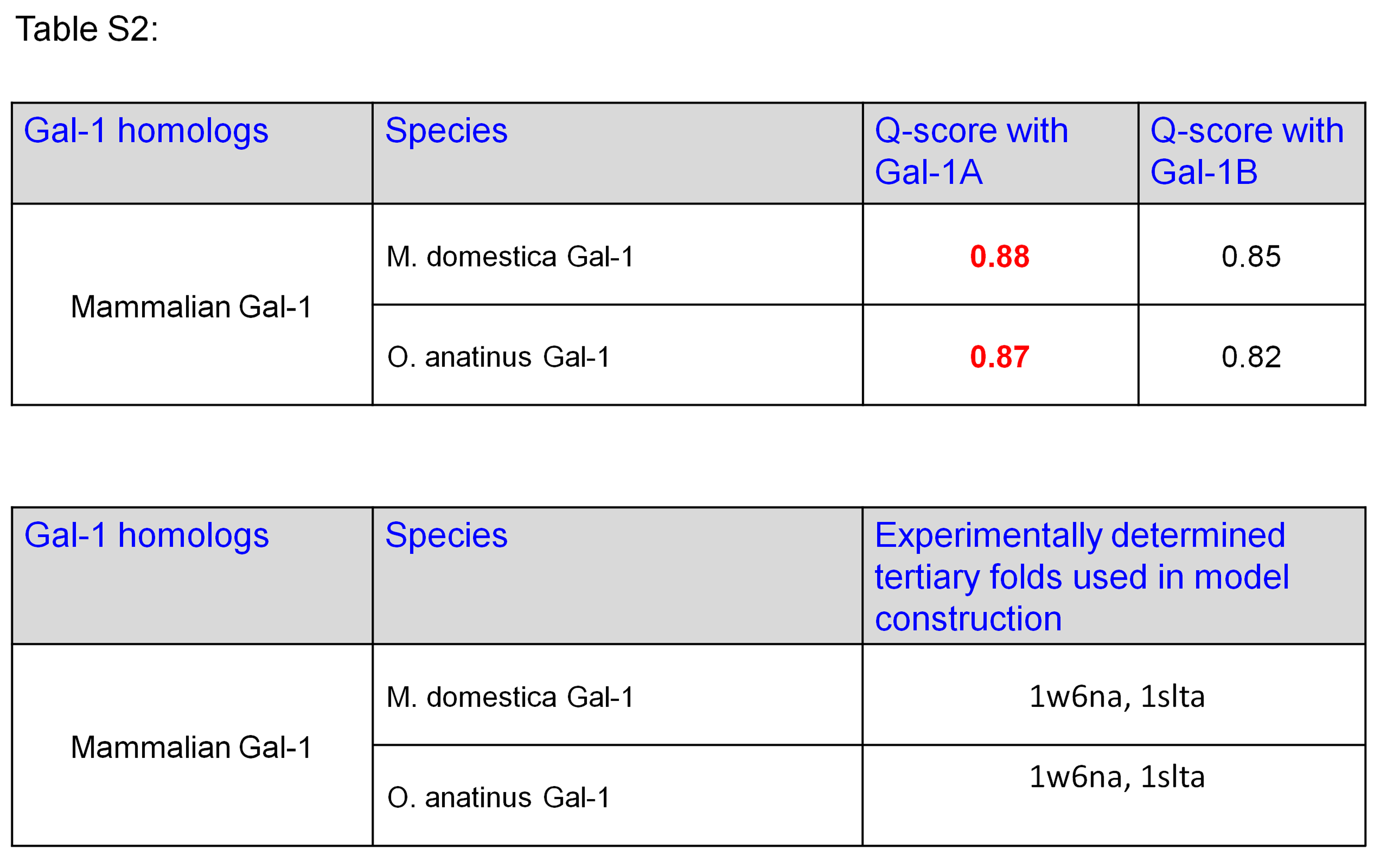

Supplement: Supplementary Data [file supp_evu215_Table_S2.tif]

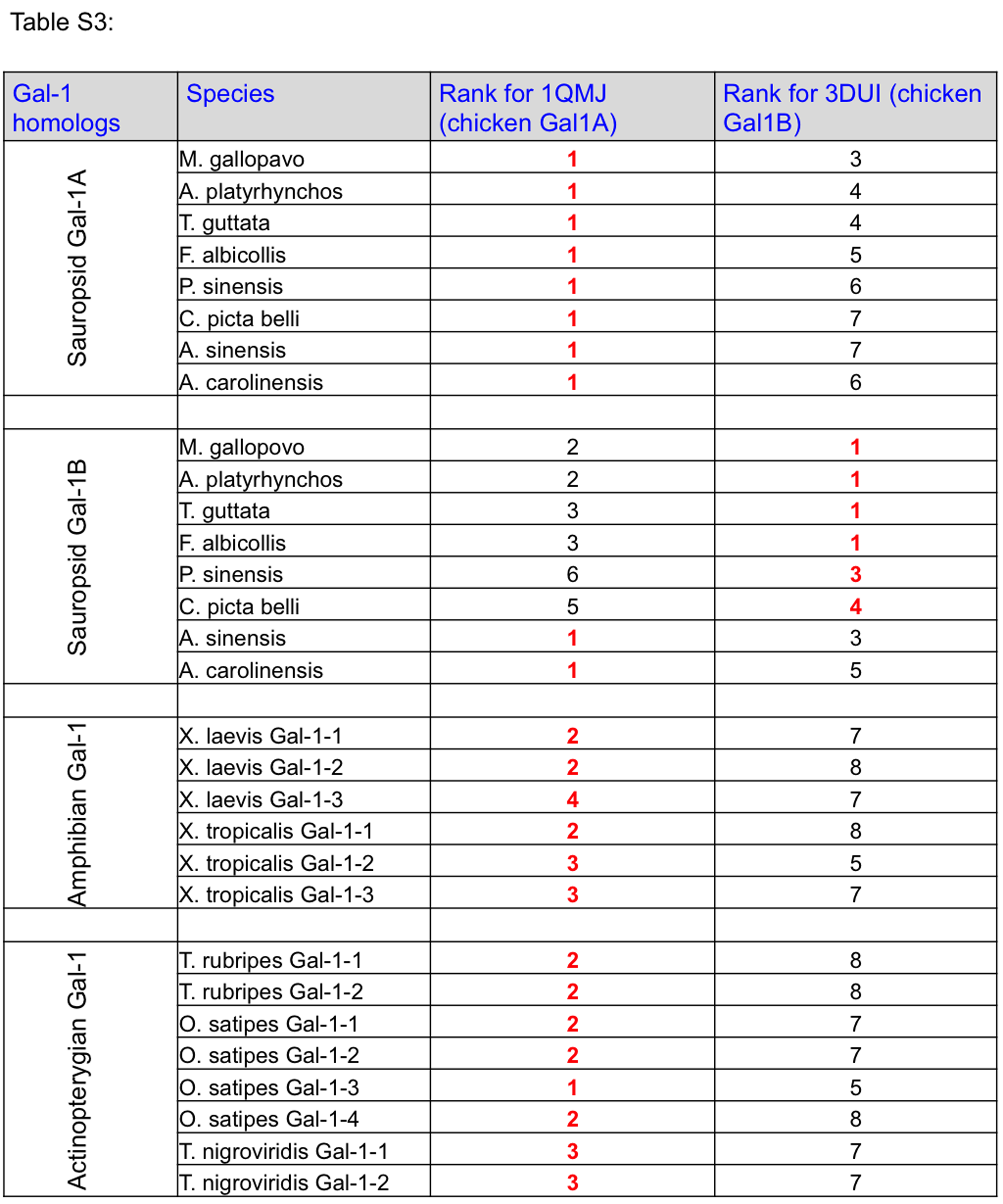

Supplement: Supplementary Data [file supp_evu215_Table_S3.tif]

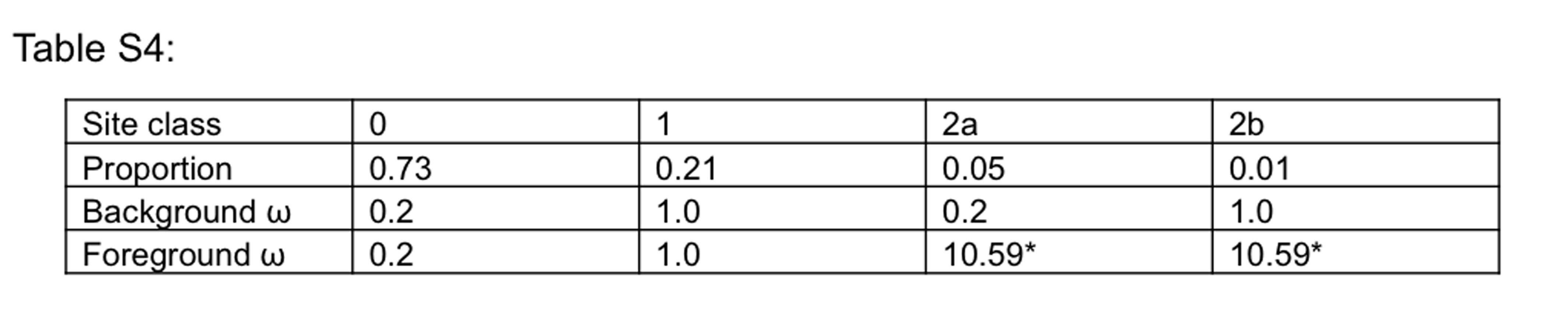

Supplement: Supplementary Data [file supp_evu215_Table_S4.tif]
